# Early biliary decompression versus conservative treatment in acute biliary pancreatitis (APEC trial): study protocol for a randomized controlled trial

**DOI:** 10.1186/s13063-015-1132-0

**Published:** 2016-01-05

**Authors:** Nicolien J. Schepers, Olaf J. Bakker, Marc G. H. Besselink, Thomas L. Bollen, Marcel G. W. Dijkgraaf, Casper H. J. van Eijck, Paul Fockens, Erwin J. M. van Geenen, Janneke van Grinsven, Nora D. L. Hallensleben, Bettina E. Hansen, Hjalmar C. van Santvoort, Robin Timmer, Marie-Paule G. F. Anten, Clemens J. M. Bolwerk, Foke van Delft, Hendrik M. van Dullemen, G. Willemien Erkelens, Jeanin E. van Hooft, Robert Laheij, René W. M. van der Hulst, Jeroen M. Jansen, Frank J. G. M. Kubben, Sjoerd D. Kuiken, Lars E. Perk, Rogier J. J. de Ridder, Marno C. M. Rijk, Tessa E. H. Römkens, Erik J. Schoon, Matthijs P. Schwartz, B. W. Marcel Spanier, Adriaan C. I. T. L. Tan, Willem J. Thijs, Niels G. Venneman, Frank P. Vleggaar, Wim van de Vrie, Ben J. Witteman, Hein G. Gooszen, Marco J. Bruno, for the Dutch Pancreatitis Study Group

**Affiliations:** Department of Gastroenterology and Hepatology, Erasmus Medical Center, PO 2040, 3000 CA Rotterdam, The Netherlands; Department of Gastroenterology and Hepatology, St Antonius Hospital, PO 2500, 3430 EM Nieuwegein, The Netherlands; Department of Surgery, University Medical Center Utrecht, PO 85500, 3508 GA Utrecht, The Netherlands; Department of Surgery, Academic Medical Center University of Amsterdam, PO 22660, 1100 DD Amsterdam, The Netherlands; Department of Radiology, St Antonius Hospital, PO 2500, 3430 EM Nieuwegein, The Netherlands; Clinical Research Unit, Academic Medical Center University of Amsterdam, PO 22660, 1100 DD Amsterdam, The Netherlands; Department of Surgery, Erasmus Medical Center, PO 2040, 3000 CA Rotterdam, The Netherlands; Department of Gastroenterology and Hepatology, Academic Medical Center University of Amsterdam, PO 22660, 1100 DD Amsterdam, The Netherlands; Department of Gastroenterology and Hepatology, Radboud University Nijmegen Medical Centre, HP 690, PO 9101, 6500 HB Nijmegen, The Netherlands; Department of Surgery, St Antonius Hospital, PO 2500, 3430 EM Nieuwegein, The Netherlands; Department of Gastroenterology and Hepatology, Sint Franciscus Gasthuis, PO 10900, 3004 BA Rotterdam, The Netherlands; Department of Gastroenterology and Hepatology, Reinier de Graaf Hospital, Reinier de Graafweg 3-11, 2625 AD Delft, The Netherlands; Department of Gastroenterology and Hepatology, VU University Medical Center Amsterdam, PO Box 7057, 1007 MB Amsterdam, The Netherlands; Department of Gastroenterology and Hepatology, University Medical Center Groningen, PO 30001, 9700 RB Groningen, The Netherlands; Department of Gastroenterology and Hepatology, Gelre Hospital, PO 9014, 7300 DS Apeldoorn, The Netherlands; Department of Gastroenterology and Hepatology, St. Elisabeth Hospital, PO 90151, 5000 LC Tilburg, The Netherlands; Department of Gastroenterology and Hepatology, Kennemer Gasthuis, PO 417, 2000 AK Haarlem, The Netherlands; Department of Gastroenterology and Hepatology, Onze Lieve Vrouwe Gasthuis, Postbus 95500, 1090 HM Amsterdam, The Netherlands; Department of Gastroenterology and Hepatology, Maasstad Hospital, Maasstadweg 21, 3079 DZ Rotterdam, The Netherlands; Department of Gastroenterology and Hepatology, Sint Lucas Andreas Hospital, PO 9243, 1006 AE Amsterdam, The Netherlands; Department of Gastroenterology and Hepatology, Medical Center Haaglanden, PO 432, 2501 CK Den Haag, The Netherlands; Department of Gastroenterology and Hepatology, Maastricht University Medical Center, PO 5800, 6202 AZ Maastricht, The Netherlands; Department of Gastroenterology and Hepatology, Amphia Hospital, PO 90158, 4800 RK Breda, The Netherlands; Department of Gastroenterology and Hepatology, Jeroen Bosch Hospital, PO 90153, 5200 ME ‘s-Hertogenbosch, The Netherlands; Department of Gastroenterology and Hepatology, Catharina Hospital, PO 1350, 5602 ZA Eindhoven, The Netherlands; Department of Gastroenterology and Hepatology, Meander Medical Center, PO 1502, 3800 BM Amersfoort, The Netherlands; Department of Gastroenterology and Hepatology, Rijnstate Hospital, PO 9555, 6800 TA Arnhem, The Netherlands; Department of Gastroenterology and Hepatology, Canisius-Wilhelmina Hospital, PO 9015, 6500 GS Nijmegen, The Netherlands; Department of Gastroenterology and Hepatology, Martini Hospital, PO 30033, 9700 RM Groningen, The Netherlands; Department of Gastroenterology and Hepatology, Medisch Spectrum Twente, PO 50000, 7500 KA Enschede, The Netherlands; Department of Gastroenterology and Hepatology, University Medical Center Utrecht, PO 85500, 3508 GA Utrecht, The Netherlands; Department of Gastroenterology and Hepatology, Albert Schweitzer Hospital, PO 444, 3300 AK Dordrecht, The Netherlands; Department of Gastroenterology and Hepatology, Hospital Gelderse Vallei Ede, PO 9025, 6710 HN Ede, The Netherlands; Department of Operating Rooms - Evidence Based Surgery, Radboud University Nijmegen Medical Centre, HP 690, PO 9101, 6500 HB Nijmegen, The Netherlands

**Keywords:** Acute pancreatitis, Treatment, Endoscopy, Pancreas, ERCP, ERC

## Abstract

**Background:**

Acute pancreatitis is mostly caused by gallstones or sludge. Early decompression of the biliary tree by endoscopic retrograde cholangiography (ERC) with sphincterotomy may improve outcome in these patients. Whereas current guidelines recommend early ERC in patients with concomitant cholangitis, early ERC is not recommended in patients with mild biliary pancreatitis. Evidence on the role of routine early ERC with endoscopic sphincterotomy in patients without cholangitis but with biliary pancreatitis at high risk for complications is lacking. We hypothesize that early ERC with sphincterotomy improves outcome in these patients.

**Methods/Design:**

The APEC trial is a randomized controlled, parallel group, superiority multicenter trial. Within 24 hours after presentation to the emergency department, patients with biliary pancreatitis without cholangitis and at high risk for complications, based on an Acute Physiology and Chronic Health Evaluation (APACHE-II) score of 8 or greater, Modified Glasgow score of 3 or greater, or serum C-reactive protein above 150 mg/L, will be randomized. In 27 hospitals of the Dutch Pancreatitis Study Group, 232 patients will be allocated to early ERC with sphincterotomy or to conservative treatment. The primary endpoint is a composite of major complications (that is, organ failure, pancreatic necrosis, pneumonia, bacteremia, cholangitis, pancreatic endocrine, or exocrine insufficiency) or death within 180 days after randomization. Secondary endpoints include ERC-related complications, infected necrotizing pancreatitis, length of hospital stay and an economical evaluation.

**Discussion:**

The APEC trial investigates whether an early ERC with sphincterotomy reduces the composite endpoint of major complications or death compared with conservative treatment in patients with biliary pancreatitis at high risk of complications.

**Trial registration:**

Current Controlled Trials ISRCTN97372133 (date registration: 17-12-2012)

**Electronic supplementary material:**

The online version of this article (doi:10.1186/s13063-015-1132-0) contains supplementary material, which is available to authorized users.

## Background

Acute pancreatitis is a leading cause for acute hospitalization [1]. In most cases, pancreatitis results from gallstones causing obstruction of Vater’s ampulla [2, 3]. As biliary stones or sludge is thought to initiate and aggravate pancreatitis, early biliary decompression – achieved by endoscopic retrograde cholangiography (ERC) with sphincterotomy – may ameliorate the disease course [4, 5]. In return, ERC with sphincterotomy itself may also be associated with complications in up to 10 % of patients [6, 7].

Several studies have investigated the effect of routine ERC in biliary pancreatitis [8]. Guidelines state an undisputed indication for ERC in patients with concurrent cholangitis and pancreatitis [2, 9]. In patients with mild disease, early ERC is not indicated because in these patients the risk for complications does not outweigh the potential benefit [2, 9].

Recent guidelines advise to perform ERC with sphincterotomy in case of pancreatitis with cholestasis, but acknowledge the moderate quality of evidence for this recommendation [2, 9]. For those patients with pancreatitis and at high risk for developing complications (that is, predicted to be severe) without cholangitis, data are conflicting. A meta-analysis found no beneficial effect of routine early ERC compared with conservative treatment [8]. However, the studies have notable shortcomings that preclude reliable recommendations on the use of ERC in patients with predicted severe biliary pancreatitis without cholangitis [10]. First, patients included were those with a low pre-likelihood of a biliary etiology, those at low risk for developing complications (that is, predicted to be mild), and those with cholangitis at presentation. Second, patient selection criteria and study endpoints (complications) varied considerably between studies and also included clinically less relevant complications such as pleural effusion or ascites. Third, the trials did not present data separately regarding liver biochemical tests, an omission that precludes performing a subgroup analysis for patients with cholestasis. Fourth, routine “early” ERC was performed during a wide time frame (48 to 72 hours after admission), which may be too late to prevent complications from severe disease or increase the risk for ERC-related complications. Fifth, the trial protocols did not specify precisely when the sphincterotomy should be performed. This resulted in many patients who underwent ERC without sphincterotomy. We believe sphincterotomy should routinely be performed during ERC to decompress the biliary duct, even in the absence of gallstones or visible sludge in the common bile duct [11–13]. Sixth, no criteria were set to guarantee that ERCs were performed by experienced endoscopists, although ERC is an intervention that requires considerable training and expertise [14, 15]. Finally, even if the data of all the available randomized trials are pooled, such analysis will still not have sufficient power to detect clinically relevant and statistically significant effects of early ERC with sphincterotomy on major complications or death in patients with predicted severe biliary pancreatitis without cholangitis [8, 16].

The APEC trial is designed to investigate whether early ERC with sphincterotomy compared with conservative treatment improves outcome in patients with biliary pancreatitis without cholangitis who are at high risk for complications.

## Methods/Design

The APEC trial is a randomized controlled, parallel group, superiority, multicenter trial. Patients with acute pancreatitis will be assessed for study eligibility within 24 hours after presentation to the emergency department. Patients with biliary pancreatitis without cholangitis and at high risk of developing severe disease are eligible for randomization. Patients are randomized to early ERC with sphincterotomy or to conservative treatment (see Additional file 1: Figure S1 and Additional file 2: Figure S2). Blinding of the patients and physicians for either treatment (ERC or conservative treatment) is unfeasible. The trial will be conducted in 27 hospitals of the Dutch Pancreatitis Study Group. The APEC trial protocol is in accordance with the Spirit Guidelines [17].

### Primary endpoint

The primary endpoint is a composite of major complications or death occurring within 180 days after randomization (that is, the composite endpoint can only occur once per patient). Major complications are defined as persistent organ failure, pancreatic necrosis, bacteremia, cholangitis, pneumonia, and pancreatic endocrine or exocrine insufficiency (see Additional file 3: Table S1 for definitions).

### Secondary endpoints

The secondary endpoints are as follows:

1. “Per protocol” analysis of the primary endpoint
2. “As treated” analysis of the primary endpoint
3. Individual components of the primary end point
4. Multivariable analysis of the primary endpoint in case of significant differences in baseline variables
5. Infected necrotizing pancreatitis
6. Need for new intensive care unit admission
7. Length of stay at intensive care unit
8. ERC-related complications (see Additional file 4: Table S2 for definitions [39-41] )
9. Cholangitis during admission
10. Number of endoscopic, radiological, and operative (re-)interventions
11. Readmission for biliary events (recurrent acute biliary pancreatitis, cholecystitis, biliary colics, or cholangitis)
12. Difficulty of cholecystectomy (as scored by Visual Analog Scale 1 to 10)
13. Quality of life (Short Form-36 and EQ5D-5 L) including quality adjusted life years (QALY)
14. Direct medical costs and direct and indirect nonmedical costs

### Inclusion criteria

Inclusion criteria are as follows:

1. Acute pancreatitis, which is defined as the presence of at least two out of the following three criteria: 1) pain in the upper abdomen, 2) serum amylase or lipase concentration > 3 times the upper limit of normal, or 3) imaging features of acute pancreatitis on computed tomography (CT) or magnetic resonance imaging (MRI) [18].
2. High risk of developing severe disease (that is, predicted to be severe) based on either one of the following criteria: Acute Physiology and Chronic Health Evaluation (APACHE II score) ≥ 8 [19] (see Additional file 5: Table S4), 2) Modified Glasgow score ≥ 3 [20] (see Additional file 6: Table S3), or 3) C-reactive protein > 150 mg/L [21, 22].
3. High probability of a biliary etiology based on at least one of the following criteria: 1) gallstones or biliary sludge on imaging (any type), 2) dilated common bile duct on imaging defined as > 8 mm in patients ≤ 75 years or > 10 mm in patients > 75 years, 3) alanine aminotransferase (ALAT) > two times the upper limit of normal (no absolute numerical value is chosen because of the multicentric design with varying upper limits among hospitals and sex-based differences in the upper limit of normal values [23–25]).
4. Ability to perform ERC within 24 hours after presentation to the emergency department and no more than 72 hours after symptom onset.
5. In case of a previous episode of necrotizing pancreatitis, patient should be fully recovered (confirmed on imaging).
6. Age ≥18 years.
7. Written informed consent.

### Exclusion criteria

The exclusion criteria include the following:

1. Cholangitis (see Additional file 3: Table S1 for definition).
2. Pancreatitis due to other causes such as alcohol abuse (more than 4 units per day), metabolic causes (hypertriglyceridemia or hypercalcemia), medication, trauma, etc.
3. Previous pancreatic sphincterotomy or needle knife precut.
4. Chronic pancreatitis (see Additional file 7: Table S5 for definition).
5. International Normalized Ratio that cannot be corrected to less than 1.5 with clotting factors or fresh frozen plasma.
6. Pregnancy.

### Randomization

Patients are randomized to early ERC with sphincterotomy or to conservative treatment (1:1 ratio) with a web-based randomization module (ALEA, Academic Medical Center, Amsterdam, The Netherlands) in random blocks of two, four or six. At randomization, patients are stratified according to the presence of cholestasis and for region of the hospital. Cholestasis is defined as a serum bilirubin > 40 μmol/L at randomization or a dilated common bile duct (defined as > 8 mm in patients ≤ 75 years or > 10 mm in patients > 75 years).

### Treatment protocol

#### Early ERC with sphincterotomy

Early ERC with sphincterotomy is performed within 72 hours after symptom onset and within 24 hours of hospital admission. A sphincterotomy is always performed irrespective of the presence of the common bile duct stones. ERC is performed by or under the direct supervision of an experienced endoscopist, which defined as a person who has done more than 400 ERCs in his or her lifetime and has performed more than 50 ERCs yearly on average in the previous 3 years. When unable to cannulate the common bile duct, even after precut sphincterotomy, the ERC procedure will be ended, and the patient will be treated conservatively. After the patient has recovered from the acute pancreatitis attack, a repeat ERC is scheduled to perform a full sphincterotomy. According to the intention-to-treat principle, these patients will be analyzed according to their original treatment allocation, that is, early ERC with sphincterotomy. In the case of incomplete stone extraction, a plastic endoprosthesis is inserted, and an elective ERC is scheduled. Antibiotics are only administered in case of contrast injection without adequate biliary drainage.

#### Conservative treatment

Patients in the conservative group are managed according to the conservative supportive treatment regimen for patients with acute biliary pancreatitis as described in the paragraph below. A rescue ERC is performed when a patient develops cholangitis (see Additional file 3: Table S1 for definition). Whenever the attending physician is in doubt concerning whether or not an ERC should be performed, the study coordinator presents the case to an expert panel. This expert panel, consisting of an independent gastroenterologist and a gastrointestinal surgeon, provides a treatment advice within 24 hours. Retained bile duct stones are removed during an elective ERC when the patient is recovered from the initial pancreatitis episode.

### General treatment regimen

Both groups are treated with intravenous infusion of fluids to ensure adequate hydration and diuresis, appropriate analgesic treatment, enteral nutrition if necessary, treatment of endocrine and exocrine pancreatic insufficiency, and a gastric tube in case of vomiting. No antibiotic prophylaxis is given. The treating physician assesses whether the patient requires intensive care monitoring or further supportive measures (for example, mechanical ventilation). All patients will undergo a contrast-enhanced CT (CECT) 5 to 7 days after hospital admission for an assessment of pancreatic necrosis. If a patient recovers quickly and is discharged within 5 days, a routine CECT will not be performed, and the disease will be considered mild. Readmission within 10 days after the initial discharge for complications related to pancreatitis is regarded as a primary admission. The timing of the cholecystectomy is determined by the treating physician depending on the patient’s condition and outcome of the pancreatitis.

### Data collection

Clinical data are collected using case record forms. At all sites, an independent monitor, unblinded to the treatment allocation, will assess the study forms, including the informed consent documents, and compare these with source documents. The in-hospital utilization of healthcare will be registered as part of the data collection. Out-of-hospital use of healthcare will be documented by self-administered questionnaires.

### Follow-up

After hospital discharge, patients are seen at the outpatient clinic and further monitored at the discretion of the physician. After 1, 3, and 6 months, patients will receive a questionnaire (SF-36, SF-HLQ, and EQ5D-5 L, respectively) [26, 27]. A visit is scheduled at 3 months after randomization to identify persistent common bile duct stones or to detect endocrine or exocrine pancreatic insufficiency (serum liver and glucose measurements and fecal elastase).

### Safety

An independent Data Safety Monitoring Committee (DSMC) has been appointed to assess protocol adherence, patient recruitment, and patient safety. All physicians who are involved in the trial are asked to report all adverse events to the coordinating investigator. Adverse events are reported using the online module (https://www.toetsingonline.nl) of the Dutch Central Committee on Research involving human subjects. All adverse events are collected and reported unblinded to the DSMC every time 60 patients are randomized, after randomization of the final patient, and at the end of follow-up of the final patient. In addition, a continuous sequential safety analysis on mortality is performed to ensure the patient’s safety throughout the trial. The DSMC discusses all adverse events and the progress of the trial and reports to the trial steering committee. A copy is sent to the ethical committee and all physicians who are involved with the study.

### Ethics

The APEC trial is performed in accordance with the declaration of Helsinki and the Dutch law regarding research involving human subjects (Wet Medisch wetenschappelijk Onderzoek met Mensen). Informed consent will be obtained from each participant. The ethical committee of the Erasmus Medical Center in Rotterdam, the Netherlands, approved the study protocol on the 12th of December 2012. Subsequently, the boards of the 27 participating hospitals gave permission for conducting the trial (see Additional file 8 Ethical bodies that approved the trial). The APEC trial is registered with identification number ISRCTN97372133.

### Statistical considerations

#### Sample size calculation

The sample size calculation is based on a recent Dutch multicenter observational study of patients with biliary pancreatitis at high risk for complications [11]. The primary endpoint occurred in 32 % of the patients in which ERC was performed compared with 46 % of the patients who were treated conservatively. Taking into account that ERC was not always performed within 24 hours and that sphincterotomy was not routinely performed, a correction factor of 2 % for both percentages is added to both incidence rates. The APEC trial is a superiority trial in which the sample size calculation is based on the assumption that early ERC with sphincterotomy reduces the incidence of the primary endpoint by 18 % (48 % to 30 %). With a power of 80 %, a two-sided significance level of 5 % and a 1 % drop-out rate, a total of 232 patients are required to be included in the study (http://www.stat.ubc.ca/~rollin/stats/ssize/b2.html, accessed 20 July 2015)).

#### Descriptive statistics

The following patient characteristics before randomization will be described: age, sex, body mass index, comorbidity, American Society of Anesthesiologists (ASA) score, duration of symptoms before randomization, duration of symptoms before ERC, serum bilirubin levels, dilated common bile duct on ultrasound or computed tomography, presence of (multi) organ failure or systemic inflammatory response syndrome (SIRS), Sequential Organ Failure Assessment (SOFA) scale [28], Multiple Organ Dysfunction Score (MODS) [29], predicted disease severity according to APACHE-II, modified Glasgow, blood urea nitrogen, and C-reactive protein. Data will be presented in percentages for categorical variables. Continuous data with a normal distribution will be presented as a mean with standard deviation and as median with interquartile range in case of skewed distribution.

#### Analyses

After 232 patients have completed their 6 months of follow-up, raw data regarding potential endpoints will be presented to an adjudication committee blinded to the treatment allocation to determine whether the endpoints meet the protocol-specified criteria. The study coordinator will blind the patient reports for treatment allocation. Each member of the committee will individually assess the potential endpoints. In case of dissenting opinions, a consensus meeting will follow. Only after consensus has been reached on each individual endpoint for each individual patient will a final analysis be performed by an independent statistician, unblinded for treatment allocation. Primary analysis, using the Pearson’s Chi-squared test, is based on the intention-to-treat principle, with patients being analyzed according to original treatment allocation, regardless of whether the cannulation or sphincterotomy was successful. For exploratory reasons a per-protocol analysis will be performed to compare treatment groups. A tabular listing of all patients excluded from the intention-to-treat populations will be provided together with the reasons for exclusion. Data will be presented as relative risks with 95 % confidence intervals. A two-tailed P < 0.05 is considered statistically significant.

#### Additional analyses

Predefined subgroup analysis will be done according to the presence of cholestasis. Logistic regression models will be used to test whether treatment effects differ significantly between these subgroups. Secondary endpoints will be compared using the Pearson’s chi-squared test or Mann-Whitney U test. Additionally, secondary endpoints will be analyzed separately using Cox regression analysis censoring patients no longer at risk and categorizing missing data as no event. For all other analyses, data will be considered missing at random. To evaluate differences in systemic inflammatory response after randomization, the APACHE-II, C-reactive protein levels, and presence of SIRS from randomization to day 7 will be calculated and compared between the treatment groups. To gain further insight into factors that are predictive of major complications or death after ERC, an exploratory analysis of the effects of (essential) baseline covariates (and potential interactions) will be performed using logistic regression analysis. The essential baseline covariates that will be studied are demographics, comorbidity, predicted severity prior to randomization, presence of organ failure prior to randomization, cholestasis, and duration of symptoms prior to randomization. In addition, the time between the start of symptoms and the ERC will be studied.

Direct medical and nonmedical costs and indirect costs will be compared to assess costs per patient with poor outcome (death or severe complications). Validated questionnaires will be analyzed to assess differences in the quality of life and provide input to compare costs per quality-adjusted life year (QALY). Health utility scoring algorithms for the EQ5D-5 L health status profiles available from the literature, based on preferences in the general population using time trade-off elicitation techniques, will be used to derive a QALY estimate for each patient. This QALY will be calculated as the product sum of health utilities and the lengths of the periods between the successive measurements [30, 31].

#### Premature termination of the study

An interim-analysis will be performed when 116 patients (50 %) have been randomized and discharged after their initial hospital admission. Raw data pertaining to potential endpoints will be presented to an adjudication committee blinded for treatment allocation to determine whether the endpoints meet the protocol-specified criteria. In case of dissenting opinions, a consensus meeting will follow. The interim-analysis will be performed by an independent statistician who reports to the DSMC. The DSMC will have unblinded access to all data when discussing the results of the interim-analysis and when reporting to the steering committee. The steering committee will decide upon continuation of the APEC trial. The Haybittle-Peto approach is used for beneficial effect, meaning that the trial will be ended using symmetric stopping boundaries at P < 0.001 [32, 33]. The trial will not be stopped for futility.

## Discussion

The APEC trial is designed to provide an answer to a persisting clinical dilemma: whether or not to routinely perform early ERC with sphincterotomy in patients with biliary pancreatitis at high risk for complications but without concurrent cholangitis. Guidelines clearly advise urgent ERC with sphincterotomy in patients with concomitant cholangitis and discard this intervention in patients with a predicted mild disease course. A recent Cochrane meta-analysis comparing routine ERC versus conservative treatment found no difference in complications and death in patients with pancreatitis at high risk for complications [8]. However, besides some notable limitations in the design of the studies included, the pooled sample size of patients with biliary pancreatitis who were at high risk for complications without concurrent cholangitis was too small to detect a difference in effect. As long as the precise role remains unclear of early ERC in biliary pancreatitis in patients at high risk for complications without concurrent cholangitis, either a potentially beneficial intervention is withheld from patients or they are exposed to a treatment from which they cannot benefit and may only suffer its potential complications. The APEC trial is the first randomized controlled trial in this particular subset of patients that is adequately powered to detect statistically significant differences in clinically relevant outcomes of early ERC and sphincterotomy. The APEC trial will also provide insights into the cost-effectiveness of routine early ERC and sphincterotomy and the amount of cost savings that can be achieved.

To date, the optimal timing of early ERC in biliary pancreatitis is unclear. Previous studies suggest that the severity of the pancreatitis is related to the duration of biliopancreatic ductal obstruction [34, 35]. These observations provide the rationale to perform an ERC and sphincterotomy early after the onset of symptoms. For this reason, albeit logistically challenging, in the APEC trial, ERC with sphincterotomy is performed within 72 hours after symptom onset and within 24 hours after hospital admission to achieve the optimal effect of the intervention.

Recent guidelines state that early ERC is probably beneficial in patients with cholestasis [2], albeit with the acknowledgement that the level of evidence is low to moderate. To investigate whether the potential beneficial effects of ERC with sphincterotomy depend on the presence of cholestasis, patients in the APEC trial will be stratified according to this baseline variable.

The primary endpoint of the APEC trial is a composite of major complications and death. One of the major complications that may occur in the course of biliary pancreatitis is pancreatic necrosis. In contrast to the definition of necrotizing pancreatitis according to the recently updated Atlanta classification, we excluded extrapancreatic necrosis alone as a major complication [18] with the rationale that extrapancreatic necrosis alone is suggested to be a separate entity in necrotizing pancreatitis and is associated with fewer complications compared with pancreatic necrosis [36].

Commonly used biochemical and radiologic predictors of biliary obstruction are unreliable in the early phase of acute pancreatitis [37]. Advanced imaging modalities such as magnetic resonance cholangiopancreatography (MRCP) and endoscopic ultrasound (EUS) have improved accuracy in detecting common bile duct stones and could therefore be applied to select patients for therapeutic ERC [38]. However, EUS is not available in all centers. Moreover, performing either MRCP or EUS within 24 hours after presentation can be challenging and may prove to be unfeasible because of limited local resources and expertise, particularly during on-call hours. The use of MRCP and EUS before ERC is also not incorporated in the APEC-trial because it is hypothesized that the beneficial effect of an early sphincterotomy might also be present irrespective of a stone or visible sludge in the CBD [11]. Hence, even if CBD stones are detected by EUS or MRCP, the question remains whether sphincterotomy in the early phase of the disease improves outcome in these patients.

The APEC trial will be performed in 27 centers that participate in the Dutch Pancreatitis Study Group. The fact that ERCs will not be performed exclusively in high volume expert centers but in hospitals nationwide by endoscopists with a predefined skill level ensures that the results of the APEC trial can be extrapolated to comparable clinical practice settings worldwide.

## Conclusion

In conclusion, the APEC trial is a multicenter randomized trial that investigates whether routine early ERC with sphincterotomy reduces the composite endpoint of major complications or death in patients with biliary pancreatitis at high risk for complications, but without cholangitis, as compared with conservative treatment.

### Trial status

The trial was registered on the 7th of December 2012 in the ISRCTN register. The first patient was randomized on the 1st of March 2013. To date, 144 patients have been randomized and inclusion is on schedule.

## Additional files

Additional file 1: Figure S1. Flowchart APEC trial according to CONSORT [43]. (PDF 6 kb) Additional file 2: Figure S2. Flowchart study protocol APEC trial. (PDF 6 kb) Additional file 3: Table S1. Definitions of the primary endpoint. (DOC 35 kb) Additional file 4: Table S2. ERC related complications. (PDF 139 kb) Additional file 5: Table S4. Acute Physiology and Chronic Health Evaluation (APACHE II score) [19]. (PDF 100 kb) Additional file 6: Table S3. Modified Glasgow score. [20]. (PDF 16 kb) Additional file 7: Table S5. Definition of Chronic Pancreatitis. (PDF 233 kb) Additional file 8: Ethical bodies that approved the trial. (PDF 185 kb)

## Abbreviations

ALAT: alanine aminotransferase
APACHE: acute physiology and chronic health evaluation
CBD: common bile duct
CECT: contrast-enhanced computed tomography
CT: computed tomography
DSMC: data safety monitoring committee
EUS: endoscopic ultrasound
ISRCTN: international standard randomized controlled trial number
MRCP: magnetic resonance cholangiopancreatography
MRI: magnetic resonance imaging
QALY: quality adjusted life year

## References

[1] Peery AF, Dellon ES, Lund J, Crockett SD, McGowan CE, Bulsiewicz WJ (2012). Burden of gastrointestinal disease in the United States: 2012 update. Gastroenterology.

[2] Tenner S, Baillie J, DeWitt J, Vege SS (2013). American College of G. American College of Gastroenterology guideline: management of acute pancreatitis. Am J Gastroenterol.

[3] Yadav D, Lowenfels AB (2006). Trends in the epidemiology of the first attack of acute pancreatitis: a systematic review. Pancreas.

[4] Opie EL (1901). The aetiology of acute haemorrhagic pancreatitis. Bull Johns Hop Hosp.

[5] Lerch MM, Saluja AK, Runzi M, Dawra R, Saluja M, Steer ML (1993). Pancreatic duct obstruction triggers acute necrotizing pancreatitis in the opossum. Gastroenterology.

[6] Andriulli A, Loperfido S, Napolitano G, Niro G, Valvano MR, Spirito F (2007). Incidence rates of post-ERCP complications: a systematic survey of prospective studies. Am J Gastroenterol.

[7] Freeman ML, Nelson DB, Sherman S, Haber GB, Herman ME, Dorsher PJ (1996). Complications of endoscopic biliary sphincterotomy. N Engl J Med.

[8] Tse F, Yuan Y (2012). Early routine endoscopic retrograde cholangiopancreatography strategy versus early conservative management strategy in acute gallstone pancreatitis. Cochrane Database Syst Rev.

[9] Working Group IAPAPAAPG (2013). IAP/APA evidence-based guidelines for the management of acute pancreatitis. Pancreatology.

[10] Schepers NJ, van Santvoort HC, Bruno MJ, Dutch Pancreatitis Study G (2014). ERCP for gallstone pancreatitis. N Engl J Med.

[11] van Santvoort HC, Besselink MG, de Vries AC, Boermeester MA, Fischer K, Bollen TL (2009). Early endoscopic retrograde cholangiopancreatography in predicted severe acute biliary pancreatitis: a prospective multicenter study. Ann Surg.

[12] Neoptolemos JP, Stonelake P, Radley S (1993). Endoscopic sphincterotomy for acute pancreatitis. Hepatogastroenterology.

[13] Venneman NG, Renooij W, Rehfeld JF, VanBerge-Henegouwen GP, Go PM, Broeders IA (2005). Small gallstones, preserved gallbladder motility, and fast crystallization are associated with pancreatitis. Hepatology.

[14] Adler DG, Baron TH, Davila RE, Egan J, Hirota WK, Leighton JA (2005). ASGE guideline: the role of ERCP in diseases of the biliary tract and the pancreas. Gastrointest Endosc.

[15] Baron TH, Petersen BT, Mergener K, Chak A, Cohen J, Deal SE (2006). Quality indicators for endoscopic retrograde cholangiopancreatography. Am J Gastroenterol.

[16] Petrov MS, van Santvoort HC, Besselink MG, van der Heijden GJ, van Erpecum KJ, Gooszen HG (2008). Early endoscopic retrograde cholangiopancreatography versus conservative management in acute biliary pancreatitis without cholangitis: a meta-analysis of randomized trials. Ann Surg.

[17] Chan AW, Tetzlaff JM, Altman DG, Laupacis A, Gotzsche PC, Krleza-Jeric K (2013). SPIRIT 2013 statement: defining standard protocol items for clinical trials. Ann Intern Med.

[18] Banks PA, Bollen TL, Dervenis C, Gooszen HG, Johnson CD, Sarr MG (2013). Classification of acute pancreatitis--2012: revision of the Atlanta classification and definitions by international consensus. Gut.

[19] Knaus WA, Draper EA, Wagner DP, Zimmerman JE (1985). APACHE II: a severity of disease classification system. Crit Care Med.

[20] Corfield AP, Cooper MJ, Williamson RC, Mayer AD, McMahon MJ, Dickson AP (1985). Prediction of severity in acute pancreatitis: prospective comparison of three prognostic indices. Lancet.

[21] Neoptolemos JP, Kemppainen EA, Mayer JM, Fitzpatrick JM, Raraty MG, Slavin J (2000). Early prediction of severity in acute pancreatitis by urinary trypsinogen activation peptide: a multicentre study. Lancet.

[22] Werner J, Hartwig W, Uhl W, Muller C, Buchler MW (2003). Useful markers for predicting severity and monitoring progression of acute pancreatitis. Pancreatology.

[23] Ammori BJ, Boreham B, Lewis P, Roberts SA (2003). The biochemical detection of biliary etiology of acute pancreatitis on admission: a revisit in the modern era of biliary imaging. Pancreas.

[24] Liu CL, Fan ST, Lo CM, Tso WK, Wong Y, Poon RT (2005). Clinico-biochemical prediction of biliary cause of acute pancreatitis in the era of endoscopic ultrasonography. Aliment Pharmacol Ther.

[25] Moolla Z, Anderson F, Thomson SR (2013). Use of amylase and alanine transaminase to predict acute gallstone pancreatitis in a population with high HIV prevalence. World J Surg.

[26] Ware JE, Sherbourne CD (1992). The MOS 36-item short-form health survey (SF-36). I. Conceptual framework and item selection. Med Care.

[27] Herdman M, Gudex C, Lloyd A, Janssen M, Kind P, Parkin D (2011). Development and preliminary testing of the new five-level version of EQ-5D (EQ-5D-5 L). Qual Life Res Int J Qual Life Asp Treat Care Rehab.

[28] Vincent JL, Moreno R, Takala J, Willatts S, De Mendonca A, Bruining H (1996). The SOFA (Sepsis-related Organ Failure Assessment) score to describe organ dysfunction/failure. On behalf of the Working Group on Sepsis-Related Problems of the European Society of Intensive Care Medicine. Intensive Care Med.

[29] Marshall JC, Cook DJ, Christou NV, Bernard GR, Sprung CL, Sibbald WJ (1995). Multiple organ dysfunction score: a reliable descriptor of a complex clinical outcome. Crit Care Med.

[30] Lamers LM, Stalmeier PF, McDonnell J, Krabbe PF, van Busschbach JJ (2005). Measuring the quality of life in economic evaluations: the Dutch EQ-5D tariff. Ned Tijdschr Geneeskd.

[31] Hakkaart-van Roijen LTS, Bouwmans CAM: Handleiding voor kostenonderzoek, methoden en standaard kostprijzen voor economische evaluaties in de gezondheidszorg. College voor zorgverzekeringen. Geactualiseerde versie 2010. [Manual for cost analysis in health care; Dutch]. Rotterdam, the Netherlands; 2011:2010.

[32] Haybittle JL (1971). Repeated assessment of results in clinical trials of cancer treatment. Br J Radiol.

[33] Peto R, Pike MC, Armitage P, Breslow NE, Cox DR, Howard SV (1976). Design and analysis of randomized clinical trials requiring prolonged observation of each patient. I. Introduction and design. Br J Cancer.

[34] Acosta JM, Rubio Galli OM, Rossi R, Chinellato AV, Pellegrini CA (1997). Effect of duration of ampullary gallstone obstruction on severity of lesions of acute pancreatitis. J Am Coll Surg.

[35] Runzi M, Saluja A, Lerch MM, Dawra R, Nishino H, Steer ML (1993). Early ductal decompression prevents the progression of biliary pancreatitis: an experimental study in the opossum. Gastroenterology.

[36] Bakker OJ, van Santvoort H, Besselink MG, Boermeester MA, van Eijck C, Dejong K (2013). Extrapancreatic necrosis without pancreatic parenchymal necrosis: a separate entity in necrotising pancreatitis?. Gut.

[37] van Santvoort HC, Bakker OJ, Besselink MG, Bollen TL, Fischer K, Nieuwenhuijs VB (2011). Prediction of common bile duct stones in the earliest stages of acute biliary pancreatitis. Endoscopy.

[38] Giljaca V, Gurusamy KS, Takwoingi Y, Higgie D, Poropat G, Stimac D (2015). Endoscopic ultrasound versus magnetic resonance cholangiopancreatography for common bile duct stones. Cochrane Database Syst Rev.

[39] Cotton PB, Lehman G, Vennes J, Geenen JE, Russell RC, Meyers WC (1991). Endoscopic sphincterotomy complications and their management: an attempt at consensus. Gastrointest Endosc.

[40] Myocardial infarction redefined--a consensus document of The Joint European Society of Cardiology/American College of Cardiology Committee for the redefinition of myocardial infarction. Eur Heart J. 2000; 21:1502–13. doi:10.1053/euhj.2000.2305. 10.1053/euhj.2000.2305 10973764

[41] Alpert JS, Thygesen K, Antman E, Bassand JP (2000). Myocardial infarction redefined--a consensus document of The Joint European Society of Cardiology/American College of Cardiology Committee for the redefinition of myocardial infarction. J Am Coll Cardiol.

[42] Schneider A, Lohr JM, Singer MV (2007). The M-ANNHEIM classification of chronic pancreatitis: introduction of a unifying classification system based on a review of previous classifications of the disease. J Gastroenterol.

[43] Moher D, Schulz KF, Altman DG, Group C (2001). The CONSORT statement: revised recommendations for improving the quality of reports of parallel-group randomized trials. J Am Podiatr Med Assoc.

