# Supplementary figures and images for: Early biliary decompression versus conservative treatment in acute biliary pancreatitis (APEC trial): study protocol for a randomized controlled trial
Source: Trials. 2016 Jan 5;17:5. doi: 10.1186/s13063-015-1132-0 (PMC4700728; doi:10.1186/s13063-015-1132-0)

**Figure S1.** Flowchart APEC trial according to CONSORT [43]

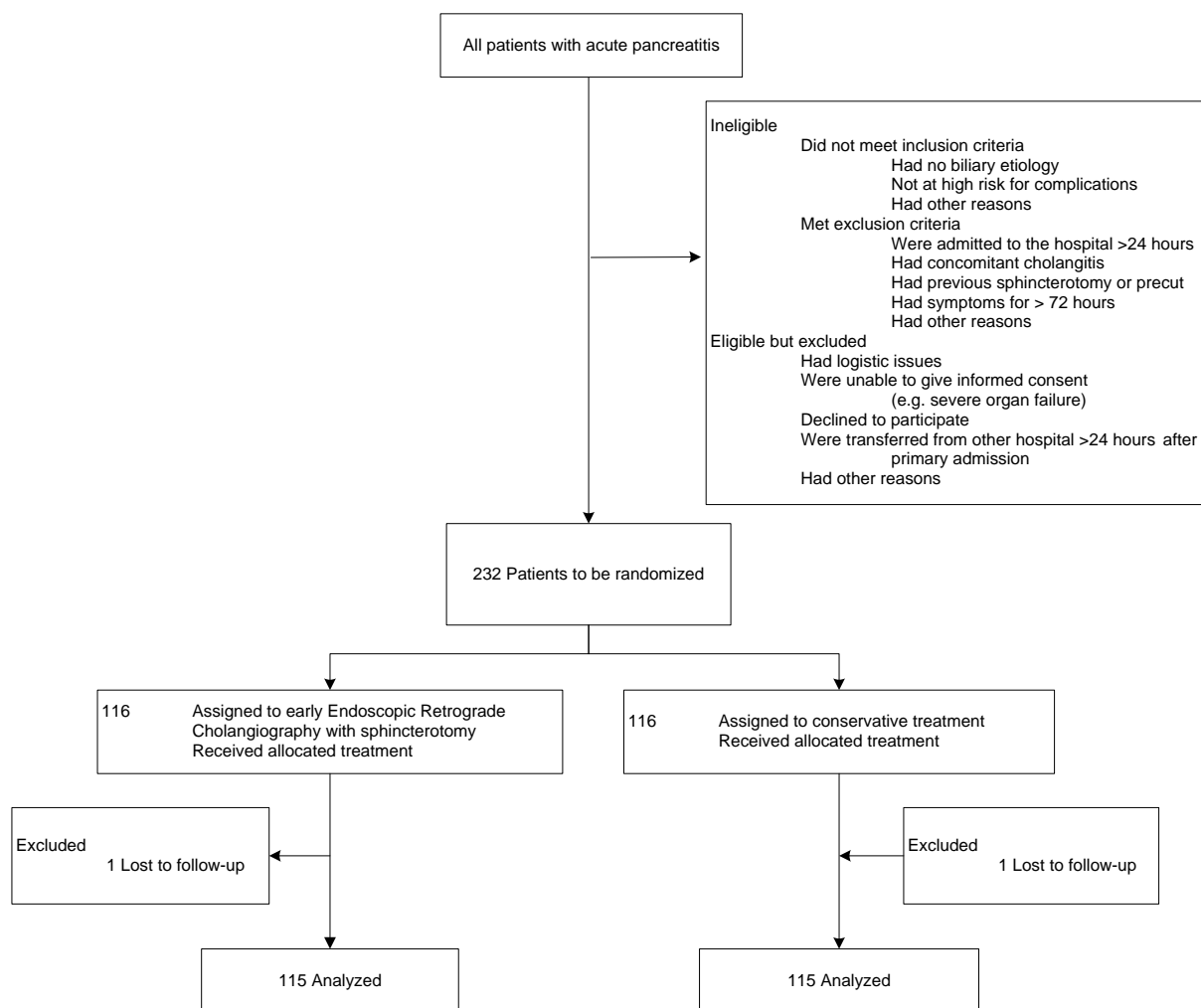

Supplement: Additional file 1: Figure S1. — Flowchart APEC trial according to CONSORT [43]. (PDF 6 kb) [file 13063_2015_1132_MOESM1_ESM.pdf]

**Figure S2.** Flowchart study protocol APEC trial

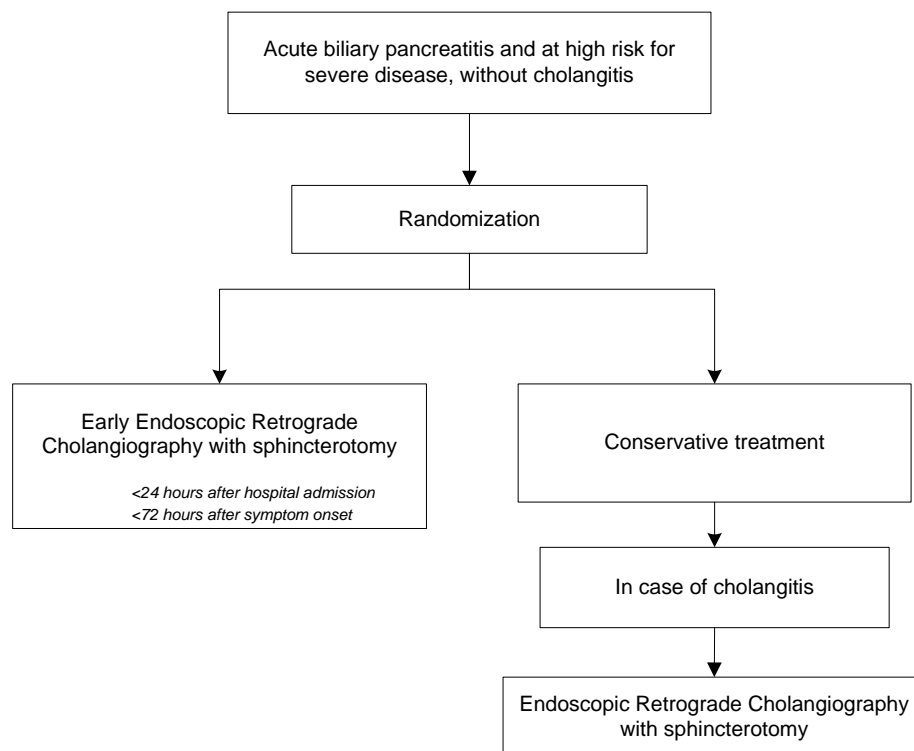

Supplement: Additional file 2: Figure S2. — Flowchart study protocol APEC trial. (PDF 6 kb) [file 13063_2015_1132_MOESM2_ESM.pdf]
