# Supplementary material for: Early biliary decompression versus conservative treatment in acute biliary pancreatitis (APEC trial): study protocol for a randomized controlled trial
Source: Trials. 2016 Jan 5;17:5. doi: 10.1186/s13063-015-1132-0 (PMC4700728; doi:10.1186/s13063-015-1132-0)
Supplement: Additional file 3: Table S1. — Definitions of the primary endpoint. (DOC 35 kb) [file 13063_2015_1132_MOESM3_ESM.doc]

**Additional file 3: Table S1**. Definitions primary end point

| **Event** | **Definition** |
| --- | --- |
| New-onset organ failure | New-onset (i.e. not present at randomisation) and persistent (i.e. >48 hours) failure of organ(s) according to the modified Marshall score . |
| Pancreatic  necrosis | Presence of diffuse or focal areas of pancreatic non-enhancement on contrast enhanced CT performed at 5-7 days after admission. |
| Bacteremia | Demonstrated with positive blood cultures. Blood samples will be drawn when temperature >38.5°C. For non-pathogens (e.g. Coagulase negative staphylococci) at least 2 samples have to be positive. |
| Cholangitis | Highest in-hospital body temperature in previous 24 hours: ≥ 38.5ºC with chills, without an obvious other cause (e.g., cystitis, pneumonia, thrombophlebitis, etc), **or** 39ºC without chills, without an obvious cause for fever, **and either:**  1) Choledocholithiasis on abdominal US, CT, EUS or MRI, **or**  2) A dilated common bile duct on imaging defined as >8mm in patients ≤75 years or >10mm in patients >75 years **or**  3) Progressive cholestasis for at least two consecutive days and a bilirubin >2.3 mg/dL (40 μmol/L). |
| Pneumonia | Coughing, dyspnoea, chest film showing infiltrative abnormalities, lowered arterial blood gas with positive sputum culture. If in intensive care, a positive endotracheal culture is mandatory. |
| Exocrine pancreatic  insufficiency | Fecal elastase <200µg/g and the need for pancreatic enzyme supplementation at 3 months after discharge; this requirement was not present before onset of pancreatitis. |
| Endocrine  pancreatic  insufficiency | The need for insulin or oral antidiabetic drugs at 3 months after discharge; this requirement was not present before onset of pancreatitis. |
