# Supplementary material for: Early biliary decompression versus conservative treatment in acute biliary pancreatitis (APEC trial): study protocol for a randomized controlled trial
Source: Trials. 2016 Jan 5;17:5. doi: 10.1186/s13063-015-1132-0 (PMC4700728; doi:10.1186/s13063-015-1132-0)
Supplement: Additional file 4: Table S2. — ERC related complications. (PDF 139 kb) [file 13063_2015_1132_MOESM4_ESM.pdf]

**Additional file 4: Table S2.** ERC related complications

| **Event** | **Definition** |
|---|---|
| Clinically relevant bleed | The presence of melena, hematochezia of hematemesis, in combination with a hemoglobin drop of 1.3 mmol/L or the need for blood transfusion (defined according to the American Society for Gastrointestinal Endoscopy ASGE [39]) |
| Perforation | New development of free gas on imaging with progressive complaints of abdominal discomfort and pain after ERC, or perforation detected at surgery |
| Respiratory insufficiency | $pO_2$<60mmHg despite $FiO_2$ of 30% or requiring mechanical ventilation |
| Cardiovascular complications | |
| ○ Acute myocardial infarction | (1) Typical rise and gradual fall (troponin) or more rapid rise and fall (CK-MB) of biochemical markers of myocardial necrosis with at least one of the following: (a) ischemic symptoms; (b) development of pathologic Q-waves on the ECG; (c) ECG changes indicative of ischemia (ST segment elevation or depression); or (d) coronary artery intervention (e.g., coronary angioplasty) [40, 41]. |
| ○ Cerebrovascular accident | Defined by the clinical event and subsequent findings on cross-sectional imaging investigations |
| ○ Shock | Systolic blood pressure below 90 mmHg despite adequate fluid resuscitation or need for inotropic catecholamine support |
