# Supplementary material for: Early biliary decompression versus conservative treatment in acute biliary pancreatitis (APEC trial): study protocol for a randomized controlled trial
Source: Trials. 2016 Jan 5;17:5. doi: 10.1186/s13063-015-1132-0 (PMC4700728; doi:10.1186/s13063-015-1132-0)
Supplement: Additional file 5: Table S4. — Acute Physiology and Chronic Health Evaluation (APACHE II score) [19]. (PDF 100 kb) [file 13063_2015_1132_MOESM5_ESM.pdf]

Additional file 5: Table S4. Modified Glasgow score [20]

Each parameter scores 1 point.	
Age	>55 years
PO2 arterial	<60 mmHg
Albumine	<32 g/L
Total calcium	<2 mmol/L
Leukocytes	>15 x 10^{9}/L
LDH	>600 U/L
Glucose (non diabetics)	>10 mmol/L
Ureum after rehydration	>16 mmol/L
