# Supplementary material for: Early biliary decompression versus conservative treatment in acute biliary pancreatitis (APEC trial): study protocol for a randomized controlled trial
Source: Trials. 2016 Jan 5;17:5. doi: 10.1186/s13063-015-1132-0 (PMC4700728; doi:10.1186/s13063-015-1132-0)
Supplement: Additional file 6: Table S3. — Modified Glasgow score. [20]. (PDF 16 kb) [file 13063_2015_1132_MOESM6_ESM.pdf]

Additional file 6: Table S3. Acute Physiology and Chronic Health Evaluation (APACHE II score) [19]

**Physiological variable**	High abnormal					Low abnormal			
	**+4**	**+3**	**+2**	**+1**	**0**	**+1**	**+2**	**+3**	**+4**
Temperature (°C)	≥ 41	39.0-40.9		38.5-38.9	36-38.4	24-35.9	32-33.9	30-31.9	≤ 29.9
Mean Arterial Pressure (mmHg)	≥ 160	130-159	110-129		70-109		50-69		≤ 49
Heart rate	≥ 180	140-179	110-139		70-109		55-69	40-54	≤ 39
Respiratory rate	≥ 50	35-49		25-34	12-24	10-11	6-9		≤ 5
Oxygenation<br>FiO2 ≥ 0.5, record $A\text{-}aDO_2$<br>FiO2 < 0.5, record PaO_2	≥ 500<br>-	350-49<br>-	200-349<br>-	-<br>-	< 200<br>> 70	-<br>61-70	-<br>-	-<br>55-60	-<br>≤ 55
Arterial pH	≥ 7.7	7.6-7.69		7.5-7.59	7.33-7.49		7.25-7.32	7.15-7.24	≤ 7.14
Serum sodium (mmol/l)	≥ 180	160-179	155-159	150-154	130-149		120-129	111-119	≤ 110
Serum potassium (mmol/l)	≥ 7	6.0-6.9		5.5-5.9	3.5-5.4	3-3.4	2.5-2.9		≤ 2.4
Serum creatinine (µmol/l)<br>Double score for AKI	≥ 302	169-301	125-168		53-124		≤ 52		
Haematocrit (%)	≥ 60		50-59.9	46-49.9	30-45.9		20-29.9		≤ 19
White blood cell count (total/mm^3)	≥ 40		20-39.9	15-19.9	3-14.9		1-2.9		≤ 0.9
15 - Glasgow coma scale									
Total Acute Physiology score (APS)									
Age points	< 44<br>45-54<br>55-64<br>65-74<br>≥ 75	0<br>2<br>3<br>5<br>6	Chronic Health points: If the patient has a history of severe organ system insufficiency or is immunocompromised, assign points as follows:<br>For non-operative or emergency postoperative patients, 5 points<br>For elective postoperative patients, 2 point						
APACHE-II score<br>(sum of APS + age points + chronic health points)			$A\text{-}aDO_2$, alveolar-arterial oxygen tension difference; AKI, acute kidney injury; FiO2, fractional inspired oxygen; PaO_2, arterial oxygen tension						
