# Supplementary material for: Early biliary decompression versus conservative treatment in acute biliary pancreatitis (APEC trial): study protocol for a randomized controlled trial
Source: Trials. 2016 Jan 5;17:5. doi: 10.1186/s13063-015-1132-0 (PMC4700728; doi:10.1186/s13063-015-1132-0)
Supplement: Additional file 7: Table S5. — Definition of Chronic Pancreatitis. (PDF 233 kb) [file 13063_2015_1132_MOESM7_ESM.pdf]

Additional file 7: Table S5. Definition of Chronic Pancreatitis

Chronic pancreatitis is confirmed according to the following criteria (adapted from the M-ANNHEIM diagnostic criteria) [42]:
• Typical clinical history of chronic pancreatitis (i.e. recurrent pancreatitis or abdominal pain), **and:**
One or more of the following additional criteria for the diagnosis of chronic pancreatitis:
• Pancreatic calcifications
• Moderate or marked ductal lesions (according to the Cambridge classification) on MRCP, CT, or EUS
• Marked and persistent exocrine insufficiency defined as:<br>o Pancreatic steatorrhea clearly relieved by enzyme supplementation, and/or<br>o Fecal elastase levels of ≤200µg/g
