# Supplementary material for: Early biliary decompression versus conservative treatment in acute biliary pancreatitis (APEC trial): study protocol for a randomized controlled trial
Source: Trials. 2016 Jan 5;17:5. doi: 10.1186/s13063-015-1132-0 (PMC4700728; doi:10.1186/s13063-015-1132-0)
Supplement: Additional file 8: — Ethical bodies that approved the trial. (PDF 185 kb) [file 13063_2015_1132_MOESM8_ESM.pdf]

Additional file 8

Ethical bodies that approved the study in the various centers, all in the Netherlands.

Albert Schweitzer Hospital, Dordrecht:

- Wetenschappelijk Onderzoek Advies Commissie (WOAC)

AMC Amsterdam:

- Medisch Ethische Toetsingscommissie (MEC AMC)

Amphia Hospital, Breda:

- Adviescommissie Mensgebonden Onderzoek Amphia (AMOA)

Canisius Wilhelmina Hospital, Nijmegen:

- Lokale Toetsingscommissie (LTC)

Catharina Hospital, Eindhoven:

- Lokale beoordelingscommissie / Medisch Ethische Toetsingscommissie (METC), Catharina Ziekenhuis Eindhoven

Erasmus Medical Center, Rotterdam:

- Medisch Ethische Toetsingscommissie, Erasmus Medisch Centrum (METC-EMC)

Gelre Hospital, Apeldoorn:

- Medisch Ethische Toetsingscommissie (METC) Gelre Ziekenhuizen

Jeroen Bosch Ziekenhuis, Den Bosch:

- Wetenschapsbureau Jeroen Bosch Ziekenhuis

Kennemer / Spaarne Gasthuis, Haarlem:

- Adviescommissie lokale uitvoerbaarheid (ACLU)

Maasstad Hospital, Rotterdam:

- Toetsingscommissie Wetenschappelijk Onderzoek Rotterdam e.o. (TWOR)

Maastricht UMC, Maastricht:

- Medisch Ethische toetsingscommissie academisch ziekenhuis Maastricht en Maastricht University (METC azM/UM) / Clinical Trial Center Maastricht (CTCM)

Martini Hospital, Groningen:

- Medisch Ethische Commissie / Wetenschappelijk Instituut

Meander Medical Center, Amersfoort:

- commissie Toetsing Wetenschappelijk Onderzoek (TWO)

Medisch Spectrum Twente, Enschede:

- Medisch Ethische Toetsingscommissie Twente (METC Twente)

Onze Lieve Vrouwe Gasthuis, Amsterdam:

- Medisch-ethische commissie - Onze Lieve Vrouwe Gasthuis (MEC-OLVG)

Reinier de Graaf Hospital, Delft:

- Board of Directors Reinier de Graaf Hospital, Delft

Rijnstate Hospital, Arnhem:

- Lokale Haalbaarheidscommissie – Rijnstate Arnhem

Sint Franciscus Gasthuis, Rotterdam:

- Medisch Ethische Toetsingscommissie / adviserende commissie wetenschap

St. Antonius Hospital, Nieuwegein:

- Research & Development (R&D) Lokale Toetsing

St. Elisabeth Hospital, Tilburg:

- Medisch Ethische Toetsingscommissie (METC Brabant) / Wetenschapsbureau Elisabeth-TweeSteden Ziekenhuis

St. Lucas Andreas Hospital, Amsterdam:

- Adviescommissie uitvoering Wetenschappelijk Onderzoek (ACWO)

UMC St Radboud, Nijmegen:

- Commissie Mensgebonden Onderzoek (CMO) Radboudumc

UMC Utrecht:

- Medisch Ethische Toetsingscommissie, Universitair Medisch Centrum Utrecht (METC-UMCU)

UMCG, Groningen:

- Medisch Ethische Toetsingscommissie, Universitair Medisch Centrum Groningen (METC UMCG)

VU University Medical Center, Amsterdam:

- Medisch Ethische Toetsingscommissie – Vrije Universiteit Medisch Centrum (METc-VUmc)

Ziekenhuis Gelderse vallei, Ede:

- BeoordelingsCommissie Wetenschappelijk Onderzoek (BCWO)
